# Aphasia partnership training: What outcomes do people with aphasia, family members and speech and language therapists expect?

**DOI:** 10.1111/1460-6984.70015

**Published:** 2025-02-20

**Authors:** Rebecca Palmer, Katerina Hilari, Carla Magdalani, Joanne Coster, Suzanne Beeke, Emma Gibbs, Helen Witts, Kate Sudworth, Caroline Jagoe, Madeline Cruice

**Affiliations:** ^1^ School of Medicine and Population Health University of Sheffield Sheffield UK; ^2^ City St Georges University of London London UK; ^3^ University College London London UK; ^4^ Sheffield Teaching Hospitals NHS Foundation trust Sheffield UK; ^5^ Derbyshire Community Healthcare NHS trust Bakewell UK; ^6^ The Aphasia Partnership Training project Patient and Public Involvement; ^7^ Trinity College Dublin Dublin Ireland

**Keywords:** aphasia, consensus, dyadic communication partner training, outcomes

## Abstract

**Introduction:**

Life with aphasia affects the whole family with shorter, less frequent conversations, frustration, reduced social networks, isolation and tension in relationships. Evidence suggests communication partner training (CPT) benefits families. However, expected improvements are poorly articulated. The Aphasia Partnership Training (APT) project aimed to identify target outcomes of a new family dyad CPT programme through persons with aphasia (PWA), family member and speech and language therapist (SLT) consensus.

**Method:**

Consensus on desired outcomes was achieved through nominal groups with 20 people with mild to severe aphasia across five groups and 10 family members of people with mild to severe aphasia across three groups, each facilitated by —two to three SLTs. Twelve CPT researchers 16 clinical SLTs with experience of CPT participated in a three‐round eDelphi to gain consensus on outcomes they perceived most likely to change. Results were triangulated using a convergence coding scheme to demonstrate agreement, partial agreement, dissonance or silence amongst the three stakeholder groups.

**Results:**

All stakeholders agreed ‘conversation’ and ‘thoughts and feelings’ were very important outcomes of APT/very likely to change (agreement). Change in ‘relationships’ was very important to family members, important to PWA and considered very likely to change by SLTs (partial agreement). Change in ‘language’ (specifically talking) was very important to PWA, but not important to family members, and SLTs were uncertain about language improvement from APT (dissonance). Each outcome construct is illustrated by specific examples generated and agreed by all stakeholder groups.

**Conclusions:**

We should aim to achieve improvements in conversation and thoughts and feelings with CPT, consider the impact on relationships and investigate the potential for language improvement (talking) as an outcome of APT. Outcome measures can be selected based on good coverage of examples generated within these constructs, ensuring they are meaningful to PWA and family members.

**WHAT THIS PAPER ADDS:**

**What is already known on the subject**

- We know that people with aphasia (PWA) and their family members often find it difficult to communicate together with wide‐reaching consequences. We know that communication partner training (CPT) benefits families as demonstrated in two systematic reviews of 56 small studies. We also know what outcomes PWA and their families want from speech and language therapy in general, but there is little clarity in the literature about what outcomes are expected from CPT specifically.

**What this paper adds to existing knowledge**

- This paper highlights the outcomes PWA, family members and speech and language therapists expect from a new CPT programme called Aphasia Partnership Training. These outcomes can be considered for all family dyad CPT programmes.

**What are the potential or actual clinical implications of this work?**

- The outcomes identified through the stakeholder consensus presented from this study can be used to set goals and monitor the success of CPT, help PWA and family members decide whether they want to participate in a communication partner intervention, and ensure that measures used to evaluate the success of CPT interventions are meaningful to PWA and their families.

## INTRODUCTION

Communication is critical to building and maintaining relationships, creating a sense of self, participating in communities and promoting emotional well‐being (Simmons‐Mackie, 2018). Aphasia is an acquired communication disorder affecting understanding, talking, reading and writing, and substantially changes the way people with aphasia (PWA) and their family communication partners communicate. Aphasia is a common consequence of stroke, with approximately 3.6 million people acquiring aphasia worldwide each year and 350 000 people living with chronic post‐stroke aphasia in the United Kingdom (Stroke Association, 2018). Conversations for PWA are often shorter, less frequent, can feel strange or cold, and couples may avoid certain topics (Croteau et al., 2020). Communication breakdown can lead to frustration, conflict and disengagement adding additional burden to family members who are often managing carer roles for PWA. Broader consequences include reduced social networks, isolation, tension in relationships and distress (Le Dorze et al., 2014). PWA have identified participation in conversation as a desired outcome of speech and language therapy, and family members want to be able to have meaningful conversations, understand how to facilitate and support communication, reduce communication breakdown, express feelings, be understood by their relatives with aphasia and feel less isolated (Wallace et al., 2017a). Systematic reviews suggest communication partner training (CPT) may benefit families living with aphasia (Simmons‐Mackie et al., 2010, 2016).

CPT is an umbrella term for a group of interventions that aim to improve communication between two individuals. CPT is a complex intervention comprising multiple components (Isaksen et al., 2018). These often include a speech and language therapist (SLT) providing education about aphasia and focussing on the individual's aphasia and communication partner's skills to help them understand what causes communication breakdown. They may also include the SLT, PWA and family members identifying communication strategies to improve their communication and reduce frustration together. Examples of communication strategies include stopping test questions (those the communication partner already knows the answer to); leaving space to listen and allow the PWA time to express themselves; having pen and paper available to encourage writing of key words; showing pictures to help clarify what is being discussed; and identifying ways of indicating frustration and the need to stop and try again later. The SLT may model strategies, invite the PWA and family members to practise these, provide feedback and encourage self‐reflection.

Findings from the systematic reviews (56 studies; Simmons‐Mackie et al., 2010, 2016) suggest that CPT is effective at improving communication activity and participation of communication partners and is probably also effective for PWA (family dyad CPT comprised 43% of reviewed literature (24/56)). However, a narrative synthesis of CPT interventions (Cruice et al., 2018) revealed a lack of clarity around essential intervention components and whilst 75% of the studies reviewed provided some justification for the intervention concept, very few were theoretically underpinned or articulated intervention components. Similarly, the evidence base poorly articulates the intended changes (i.e., outcomes) for recipients from family dyad CPT leading to little clarity and consensus about what the intervention aims to achieve or how to best measure outcomes of CPT. This is illustrated by the fact that 56 different outcome measures were used across studies in the systematic reviews (Simmons‐Mackie et al., 2010, 2016). Most studies measured communication activity and participation (with different interpretations), and less than 10% studies measured psychosocial outcomes or quality of life. Methods differed widely including conversation analysis, self‐report of communication use, patient‐reported outcome measures and interviews.

Lack of understanding about outcomes of CPT is problematic. Outcomes are required to complete a programme theory of how CPT works, providing a goal for the intervention components and their mechanisms of action which ensure that outcomes are likely to be achieved in research and clinical practice alike (Sidani & Sechrest, 1999). Simmons‐Mackie et al. (2016) recommended that well‐designed trials of CPT need to be conducted to strengthen recommendations to offer this intervention. It is therefore important to understand what outcomes are intended in order to select appropriate outcome measures to evaluate the effectiveness of the intervention. Furthermore, expected outcomes need to be articulated by SLTs to families when offering intervention, so that families can decide whether CPT is likely to meet their needs and whether they wish to engage with it.

Wallace et al. (2017a) found priority outcomes from speech and language therapy for patients and families included (1) improved communication; (2) increased life participation; (3) changed attitudes through increased awareness and education about aphasia; (4) recovered normality; (5) improved physical and emotional well‐being; and (6) improved health (and support) services. These priorities are linked to all World Health Organization (WHO) ICF components: body functions, activity, participation and environmental factors (World Health Organisation, 2001). Speech and language therapy often comprises multiple interventions either delivered in combination or sequentially in order to achieve the aforementioned range of outcomes. CPT is one subtype of intervention for PWA and as yet there is no agreement about the specific outcomes desired/expected amongst SLTs who deliver it, PWA and family members who receive it.

The study reported in this paper is part of the Aphasia Partnership Training (APT) programme of research, creating a novel, well‐specified family dyad CPT intervention. The first part of the study identified core components of APT.

The second part, reported in this paper, aimed to identify the most important outcomes of APT to PWA, family members and SLTs.

Identification of outcomes enabled completion of a programme theory which will underpin further work identifying corresponding outcome measures (reported elsewhere). Outcomes were identified from an outline of APT generated from the components identified in the first part of the study. This is the first time that outcomes have been identified for a CPT intervention a priori and in tandem with developing the intervention. The outcomes identified for APT are also applicable more broadly to other dyadic CPT programmes for PWA and their family members.

## MATERIALS AND METHODS

### Study design

The study used a nominal group technique (Murphy et al., 1998) to generate and gain consensus on important outcomes of APT from the perspective of PWA and family members, and an eDelphi to gain consensus on important outcomes from SLTs. Nominal group technique was chosen as the structured turn‐taking process facilitates PWA to contribute easily, allowing facilitators to use communication skills and strategies to support the understanding and expression of ideas by individuals with different communication disorder profiles and severities. This technique was used successfully in the PWA and family member consensus on outcomes of speech and language therapy for aphasia (Wallace et al., 2017a). EDelphi for SLTs enabled wide geographical participation and collected data in a time efficient way for this stakeholder group (Wallace et al., 2017b). Ethics permission for conducting this study was received from [redacted for blind review] Ethics Committee on 22 February 2022.

### Nominal groups

#### Participants

PWA were eligible for participation in a nominal group if they were aged 18 years or over; had a diagnosis or self‐report of aphasia as a result of stroke(s) confirmed by the SLT research assistant or local SLT co‐investigator; were able to read and understand spoken language at a two key word level minimum, produce some intelligible spoken output and/or write single key words as determined by the Consent Support Tool (Palmer & Jayes, 2016); had experience of National Health Service (NHS) treatment for aphasia (as the outcomes were being identified for a new CPT programme designed predominantly for delivery in the NHS); had a regular family communication partner (communicates at least three times weekly); and were able to communicate in English (language used in the nominal groups) with appropriate communication support.

PWA were excluded if aphasia was severe as determined by the Consent Support Tool (less than two key word comprehension and/or reading ability, and/or no intelligible verbal output); aphasia was very mild determined by a score of 5 on the Boston Diagnostic Aphasia Severity Rating Scale (Goodglass et al., 2001); individuals had moderate or severe comorbid cognitive impairment (identified through discussion with a family communication partner), and/or moderate or severe self‐reported neurological conditions other than stroke (e.g., Parkinson's/Motor Neurone Disease).

Family members were eligible to participate in nominal groups if they were aged 18 years or over; and were the family communication partner of someone diagnosed with poststroke aphasia (self‐reported by completion of the Carer Communication Outcomes After Stroke questionnaire (Long et al., 2009); their family member with aphasia received NHS speech and language therapy (self‐reported); had frequent communication with their family member with aphasia (at least three times weekly); and were able to communicate in English in the nominal groups.

Family members were excluded if they reported their relative with aphasia had moderate or severe cognitive impairment, and/or moderate or severe other neurological conditions (e.g., Parkinson's/Motor Neurone Disease).

As the APT intervention was being developed initially for delivery in the UK NHS, nominal groups were held in the United Kingdom in different areas to maximise diversity of participants who had received NHS speech and language therapy (rural/urban, ethnicity). PWA and family members were therefore recruited from voluntary stroke and aphasia support groups in South Yorkshire, Derbyshire and London. Potential participants were identified by the SLT research assistant or a local SLT co‐investigator attending voluntary group meetings or events or providing information for voluntary group facilitators to distribute. Potential participants could self‐identify by responding to study advertisements in the public domain (e.g., via social media). In addition, research team members contacted PWA and family members who previously consented to be contacted about new research.

All potential participants were provided with written information about the nature and objectives of the study and given at least 24 h to decide whether to take part. Aphasia‐friendly recruitment materials and processes were prepared with the patient and public involvement (PPI) group in line with the Consent Support Tool (Palmer & Jayes, 2016). The Consent Support Tool's communication screening test was used with PWA by the SLT research assistant or local SLT co‐investigator once a potential participant had demonstrated interest in the study to identify the individual's communication profile and provide the information in the most appropriate way (written lay language;  written information using the full range of aphasia accessible principles; or a PowerPoint presentation delivered  by the research assistant or SLT co‐investigator, both experienced in supporting communication difficulties). Potential participants were given the opportunity to discuss the research with the research team and ask questions. Formal written consent was obtained for all research participants, the process for which was adapted to allow those participants unable to physically write to type in their name electronically or nominate a family member to sign on their behalf. Once a participant had consented to be part of a nominal group, the recruiting researcher or SLT collected demographic information including age, gender, time since aphasia onset, ethnicity, languages spoken and most frequent communication partner. A judgement of aphasia severity was also made by the recruiting SLT/researcher based on the outcome of the Consent Support Tool screening test which had been used to identify the appropriate information style to use to explain the study. For family members, the severity of aphasia of their communication partner was determined between the family member and the recruiting SLT/researcher using the Boston Diagnostic Aphasia Examination severity rating scale.

Twenty PWA attended five nominal groups (two in Derbyshire for those recruited from Derbyshire and neighbouring county, South Yorkshire and three in London), and 10 family members^1^ attended two nominal groups (one in Derbyshire, one in London). Participants were allocated to groups based on their availability for attendance, geographical proximity and ensuring that there were more than three but no more than six in each group, as Aspinal et al. (2006) and Vella et al. (2000) reported increased difficulty prioritising ideas generated with increasing group numbers in their nominal group studies. PWA and family member characteristics are shown in Table 1.

**TABLE 1:** PWA (*n* = 20) and family members (*n* = 10) participant characteristics.

Characteristic		PWA	Family members
		Number (%)	Number (%)
Age (range 43–79 years)	Younger than 65 years	11 (55)	5 (50)
	65 years or older	6 (30)	5 (50)
		*Nb. 3 missing data points*	
Gender	Male	14 (70)	3 (30)
	Female	6 (30)	7 (70)
Aphasia severity (of PWA participant or family member's communication partner)	Mild	10 (50)	6 (60)
	Moderate	8 (40)	2 (20)
	Moderate/severe	2 (10)	1 (10)
			*Nb. 1 missing data point*
Time since aphasia onset (of PWA participant or family member's communication partner)	1‐2 years	4 (20%)	3 (30)
	3–5 years	4 (20%)	3 (30)
	6–10 years	5 (25%)	2 (20)
	11 years or more	5 (25%)	2 (20)
		*Nb. 2 missing data points* (range 1–16 years)	(range 2–19 years)
Geographical location	South Yorkshire/Derbyshire	9 (45)	6 (60)
	London	11 (55)	4 (40)
Ethnicity	White English/Scottish/Welsh/Northern	13 (65)	10 (100)
	Irish/UK	3 (15%)	
	White—Irish	1 (5%)	
	White—Other	2 (10%)	
	Black—African	1 (5%)	
	Black—British	1 (5%)	
	Arabic	*Nb. 1 participant identified as having two ethnicities*	
Languages spoken	Monolingual—English only	12 (60)	6 (60)
	Bilingual—English + 1 other language	3 (15)	3 (30)
	Multilingual—English plus 2 or more other languages	4 (20)	1 (10)
	Languages spoken:	English, Arabic, Broken English (Nigeria) French, Gaelic, German, Hausa, Hebrew, Polish, Russian and Yoruba.	English, French, German and Spanish
		*Nb. 1 missing data point*	
Person PWA most frequently communicates with	Spouse/partner	12 (60)	9 (90)
	Parent(s)	5 (25)	
	Child(ren)	14 (70)	1 (10)
	Sibling(s)	9 (45)	
	Grandchild(ren)	6 (30)	
	Other family members	8 (40)	
Family member relationship to communication partner with aphasia			

Abbreviation: PWA, people with aphasia.

### Procedure

#### Group facilitation

Each group was facilitated by at least two registered SLTs, at least one of whom was also a researcher. The therapists used supported communication techniques to facilitate participation of PWA for example, working one to one, paraphrasing ideas, using gesture, writing, drawing and picture material. To ensure consistency across groups, the same SLT research assistant was present at all groups and a detailed facilitator guide was provided to each facilitator and discussed prior to each group.

#### Scene setting and the nominal question

The groups started with an accessible written and verbal description of likely components making up APT derived from earlier project work and refined by the PPI group (see Appendix 1). Participants were asked to imagine having APT as part of their speech and language therapy.

The nominal question, developed with the project PPI group ‘What improvements would you hope APT would make?’ was then posed to the group verbally and in writing.

#### Constructs, item generation and grouping

The nominal group technique steps of independent idea generation followed by sharing of ideas (round robin) were adhered to using accessible resources. The outcome ideas (items) were grouped into the pre‐defined constructs, informed by the WHO ICF framework: ‘talking’ (body functions/impairment), ‘conversation’ (activity), ‘doing things’ (participation), ‘thoughts and feelings’ (well‐being), ‘relationships’ (well‐being) and ‘other’ construct headings were generated.

#### Rating of constructs and items

The final step, voting, was conducted firstly at the construct level with each participant given five votes (sticky dots) to allocate across constructs; and then within each construct at the item level with three votes (dots) each. Any new ideas suggested after this point were discussed, allocated to a construct and voted on by raising hands if participants felt it was important.

### eDelphi

#### Participants

SLTs were eligible to participate in the eDelphi if they had worked in the UK NHS as a Health Care Professions Council‐registered SLT with patients with poststroke aphasia and/or family communication partners of adults with poststroke aphasia in the last 12 months, had a minimum of 12‐months experience practising as a SLT with adult neurology patients, and had experience of delivering any form of CPT to at least two patients with poststroke aphasia and/or family communication partners. Academic SLTs internationally were also invited to participate in the eDelphi if they had published at least one peer‐reviewed article on family CPT intervention in aphasia or were currently researching family CPT intervention with PWA and/or family communication partners. These professional participants had completed an eDelphi for the first stage of the APT project, so were familiar with this method.

The eDelphi survey was advertised through national and international speech and language therapy and aphasia organisations and special interest groups. Authors of publications about CPT were also emailed separately and the survey was promoted through project social media accounts. Study information and consent to participate was provided at the start of the round one questionnaire and eligibility was checked through responses to written questions. Demographic information, see Table 2, was also collected at the start of round one.

**TABLE 2:** eDelphi participants’ (*n* = 28) characteristics.

Characteristic		NHS SLTs delivering CPT (*n* = 16)	CPT researchers (*n* = 12)
		Number (%)	Number (%)
Main work setting	University		11 (92)
	Acute/subacute	1 (6%)	1 (8)
	Inpatient rehabilitation	5 (31%)	
	Community	6 (38%)	
	Outpatient rehabilitation	0 (0%)	2 (17)
	Early supported discharge	1 (6%)	
	Not‐for‐profit organisation	1 (6%)	
	Nursing homes		
	Private practice	2 (13%)	
		0 (0%)	2 (17)
Clinical educational background	Speech and language pathologist/therapist	16 (100)	11 (92)
	Linguist		1 (8)
	Psychologist		1 (8)
Number of years working with aphasia	2–5 years	1 (6)	
	6–10 years	4 (25)	
	11–15 years	5 (31)	
	16–20 years	3 (19)	
	Over 20 years	3 (19)	
Ethnicity	White	16 (100)	12 (100)
Gender	Female	16 (100)	12 (100)
Age	29 years or less		1 (8)
	30–39 years	8 (50)	2 (17)
	40–49 years	5 (31)	3 (25)
	50–50 years	3 (19)	5 (42)
	60 years or more		1 (8)
UK work region	Midlands/East England	3 (19)	
	North England	4 (25)	
	Wales	1 (6)	
	Greater London	2 (13)	
	South East England	5 (31)	
	South West England	1 (6)	
Country of residence	Brazil		1 (8)
	Canada		1 (8)
	Croatia		2 (17)
	Denmark		1 (8)
	Germany		1 (8)
	Netherlands		1 (8)
	New Zealand		1 (8)
	Norway		1 (8)
	Sweden		3 (25)
Target(s) of intervention when delivering CPT for family dyads	Person with aphasia only		1 (8)
	Family communication partner only		5 (42)
Working with	Both the person with aphasia and their family communication partner		9 (75)
Languages used to deliver CPT in	Croatian		2 (17)
	Danish		1 (8)
	Dutch		2 (17)
	English	16 (100)	3 (25)
	French		1 (8)
	Portuguese		1 (8)
	Swedish		3 (25)
	Spanish	1 (6)	
	Yiddish	1 (6)	

Abbreviations: CPT, communication partner training; NHS, National Health Service; SLT, speech and language therapist.

### Procedure

EDelphi questionnaires were prepared using Qualtrics. In round one, the participants were presented with a written description of APT. They were asked to rate the likely change in each of the same constructs as used in the PWA and family members nominal groups (‘talking’ was described as ‘language’ for SLTs) on a nine‐point Likert scale from 1 ‘not at all’ to 9 ‘a lot’. They were then asked to generate specific changes expected under each construct. After round one, the researchers conducted a thematic analysis on the specific expected changes generated where high level themes were identified inductively corresponding to constructs, and subthemes emerged from the data deductively and formed items. Researcher 1 coded all of the data and researcher 2 coded 10% of the data achieving 71% agreement. Disagreements were resolved through discussion. A unique list of items was sent out in round two for participants to rate on a nine‐point Likert scale for likelihood of change as a result of APT. A consensus was reached that a construct or item was ‘likely to change’ if an average score of 7–9 was reached. A consensus that an item was ‘unlikely to change’ was reached if the average score was 1–3 and it was considered inconclusive (no consensus reached) if the average score was 4–6. Items that did not reach consensus were sent out for re‐rating in a third eDelphi round (it was still possible to rate from 4–6 to confirm uncertainty). This approach was used in order to be able to grade the relative importance of different constructs and items.

### Analysis

#### Outcome constructs (talking/language, conversation, thoughts and feelings, relationships, doing things, other)

*Nominal groups*: The total votes for each construct across all the groups were summed for PWA and for family members separately, and the constructs were ranked according to the number of votes they received for PWA and for family members separately. The top three ranked constructs or any construct that had 10 or more votes for PWA, or 5 or more votes for family members were considered ‘very important’. Constructs that were not ranked in the top 3 but had 7– 9 votes for PWA, or 4 votes for family members, were considered ‘important’. Constructs that were not ranked in the top 3 and had 6 or fewer votes for PWA, or 3 or fewer votes for family members, were considered ‘not important’. (Note: relationship between numbers of votes and level of importance differs between PWA and family member groups due to the lower number of family member participants).

*eDelphi*: For SLTs, the level of likelihood of change was indicated by the average Likert scores. The three constructs with the highest average scores, or any construct that had an average score of 7 or more from round 1 of the eDelphi were considered as having the potential to ‘change a lot’. Any construct that was not in the top three but had an average of 4 or above was considered to have the potential to ‘change a bit’ and any construct that had an average below 4 was considered to ‘not have much potential to change’.

*Triangulation*: The levels of importance and likelihood of change were used to triangulate the construct consensus from each stakeholder group based on Farmer et al. (2006) triangulation protocol. A convergence framework was developed to indicate relative agreement about the importance/likelihood of change in an outcome construct as follows:

Agreement—Same level of importance/likelihood of change for all stakeholder groups.

Partial agreement—Same level of importance/likelihood of change for any two stakeholder groups.

Silence—Only one group suggested the construct (relevant for PWA and family members only, as SLTs were given predefined constructs to rate, but did not generate them).

Dissonance—Opposite levels of importance/likelihood of change between at least two groups.

#### Outcome items (specific examples of constructs generated by participants)

*Nominal groups*: To achieve one consensus result for PWA and one for family members on important outcome items, a qualitative content analysis was conducted using the four‐step process described by Bengtsson (2016): (1) decontextualisation, (2) recontextualisation, (3) categorisation, and (4) compilation. We employed both manifest analysis by considering what had been said/the wording of the item (surface structure) and latent analysis by considering the intended meaning (deep structure). In step 1, the items were decontextualised from the construct they had been allocated to in the consensus activity and recontextualised in step 2 by reallocating some items (meaning units) to different constructs from their original allocation. As some similar item descriptions were allocated to different constructs by different groups, steps 1 and 2 ensured all similar meaning units were allocated to the same construct to enable comparisons across stakeholder groups. Step 2 also involved removing items (meaning units) that had received no votes in the consensus activity. Step 3 ‘categorisation’ was a key stage in the process of producing one list of outcome items for PWA and one for family members. The items (meaning units) generated by each group of PWA were jointly reviewed by two research SLTs and items similar in meaning were combined and condensed into one item. Stage 4 ‘compilation’ involved the researchers naming the condensed items, keeping as close as possible to the wording used in the original items from different groups. The same process took place for items generated by family members. Modified member checking was conducted by the PPI advisory group (PWA and family members, two of whom participated in the nominal groups and four who did not) who reviewed the raw data and checked that the meaning of items which had been condensed was sufficiently similar. They also ensured that meaning from original items generated in groups was maintained where condensed. Votes for each item were then summed to indicate their relative importance.

*eDelphi*: The list of outcome items for SLT stakeholders was that generated from round 1 of the eDelphi, with relative likelihood of change indicated by the average score on the Likert scale (allocated in round 2 and 3) in the same way as for the constructs.

*Triangulation*: To achieve consensus across the three stakeholder groups, researchers grouped items that were similar in meaning across the groups, repeating stage 3, categorisation (Bengtsson, 2016). Stage 4 (compilation) was then repeated with researchers providing a summary label for the similar items across stakeholder groups. Modified member checking by the PPI group was conducted as above to maintain credibility of the resulting item labels. The items were triangulated across stakeholders based on similarity of meaning and a convergence protocol was developed to determine level of agreement on the importance of each unique labelled item (Farmer et al., 2006):

Agreement—All three groups suggested similar items, and at least one of the similar SLT items rated as likely to change a lot.

Partial agreement—(I) All three groups of participants suggested similar items BUT none rated as likely to change a lot by SLTs; (II) similar items were suggested by any 2 groups (not all 3), (III) weak similarity across groups.

Silence—Only one group suggested the item.

Dissonance—Opposing ideas in terms of meaning between groups.

## RESULTS

**FIGURE 1:**
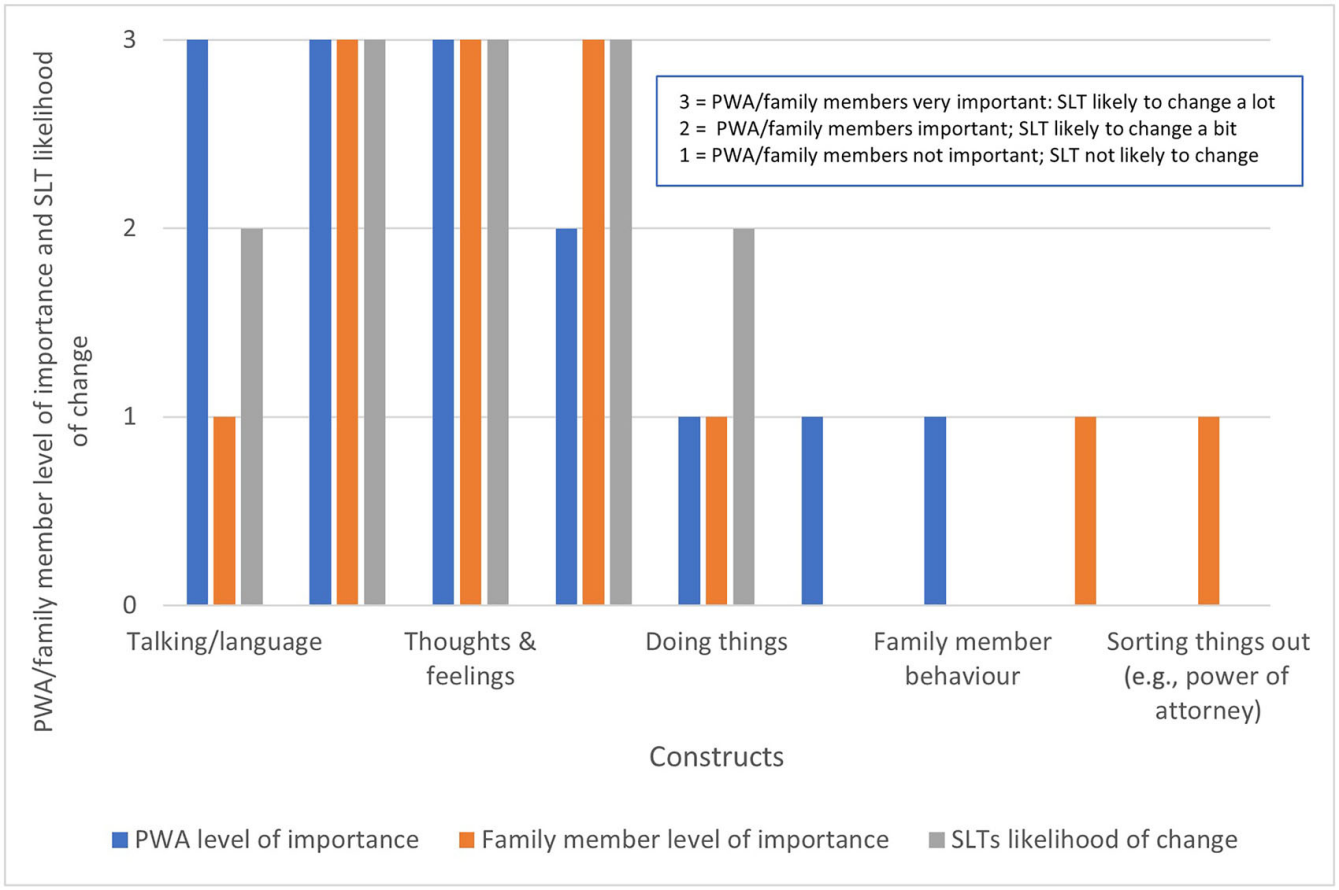
Pictorial representation of the relative importance/likelihood of change in outcome constructs. Abbreviations: PWA, people with aphasia; SLT, speech and language therapist.

Table 3 shows agreement between all stakeholder groups that ‘conversation’ and ‘thoughts and feelings’ were very important outcome constructs of APT and likely to change a lot, with ‘conversation’ being ranked higher. The impact of APT on ‘relationships’ was very important for family members and similarly, SLTs thought ‘relationships’ were likely to change a lot with APT. Impact on ‘relationships’ was important for PWA, but not as important for PWA as ‘talking’. Dissonance was identified between stakeholder groups for the impact of APT on ‘talking’ (language). Improvement in ‘talking’ was very important as an outcome of APT for PWA but not important for family members and SLTs did not reach consensus on whether it was likely to change from APT. ‘Doing things’ was not considered to be an important outcome of APT for either PWA or family members and consensus on the likelihood of this changing was not achieved for SLTs. Both PWA and family members suggested other constructs, but there was no consensus on them being priority outcomes of APT. The relative importance/likelihood of change of outcome constructs is depicted in Figure 1.

**TABLE 3:** Triangulation of relative importance/likelihood of change in outcome constructs.

	PWA level of importance	FM level of importance	SLTs likelihood of change	
Construct	(Rank: votes)	(Rank: votes)	(Rank: average Likert score)	Convergence code
Talking (language)	Very important	Not important	Likely to change a bit	Dissonance
	(1st: 22)	(5th: 1)	(5th: 4.4)	
Conversation	Very important	Very important	Likely to change a lot	Agreement
	(2nd: 17)	(1st:10)	(1st:7.6)	
Thoughts and feelings	Very important	Very important	Likely to change a lot	Agreement
	(3rd: 12)	(2nd: 9)	(2nd: 7.2)	
Relationships	Important	Very important	Likely to change a lot	Partial agreement
	(4th: 8)	(3rd: 5)	(3rd: 7.2)	
Doing things	Not important	Not important	Likely to change a bit	Partial agreement
	(7th:0)	(5th:1)	(4th:6.4)	
Taking more responsibility	Not important			Silence
	(5th:1)			
Family member behaviour	Not important			Silence
	(5th:1)			
More awareness		Not important		Silence
		(5th:1)		
Sorting things out (e.g., power of attorney)		Not important		Silence
		(4th:3)		

Abbreviations: FM, family member; PWA, people with aphasia; SLT, speech and language therapist.

Summary labels for items that stakeholders agreed (or partially agreed) were important and likely to change are used to illustrate the changes expected within each construct following APT. The triangulation exercise, including items from each stakeholder group, summary labels for groups of items across stakeholders and their respective convergence codes are detailed in Appendix 2, Tables 1a–e. Of the 12 items suggested only by one stakeholder group (silence), nine of these were identified as possible outcomes by SLTs but not by PWA or family members. As the aim of the study was to achieve consensus on outcomes, items only suggested by one stakeholder group (silence) are not discussed in the results section but are reported in Appendix 2 for completeness. Whilst there was agreement, partial agreement and some silence in the items generated by different stakeholder groups, there was no dissonance.

### Talking (language)

Table 4 shows that there was no agreement on any items illustrating expected changes in ‘talking (language)’ from APT. However, all stakeholder groups suggested that *‘the person with aphasia would be able to speak better/find their words more easily’* despite SLTs not reaching consensus on the likelihood of seeing this change.

**TABLE 4:** Summary item labels for items with agreement and partial agreement.

	Outcome constructs important to at least one stakeholder group			
Items with agreement or*partial agreement illustrating the construct in the context of APT	Talking (language)	Conversation	Thoughts and feelings	Relationships
	*The person with aphasia would be able to speak better/find their words more easily	*My family member, other people and I will use different ways of communicating that help conversation. We will be more mindful of the things we do in conversation, and work towards reducing behaviours that do not support conversation*. *Being aware or recognising the needs of self and/or of the communication partner and knowing how to communicate better together*. *Communicating more often together/with others* *Understanding more of the conversation/ understanding each other better Having a ‘decent’, more interesting or more balanced conversation* *‘*more open and honest conversations’*(partial agreement I) *‘conversations are more light‐hearted/humorous’ (partial agreement II, family members & SLTs) * ‘*Using better and faster ways of communicating (inc. non‐verbal) to repair conversation breakdowns’* (partial agreement II PWA & SLT) *Making a deliberate effort to take part in conversation (partial agreement III PWA & SLTs) *family dyad doesn't give up when conversation gets hard’ (partial agreement II family members & SLTs)	*Reduced frustration* *Feeling good, more relaxed, happy, fulfilled, free to be myself, more confident, comfortable, motivated, empowered, hopeful, optimistic, more competent* *Feeling less angry, resentful, guilty* *More able to cope* *Feeling *more normal/like themselves again* (partial II, PWA & SLTs) *reduced anxiety (partial II, PWA & SLTs) *reduced loneliness/isolation (partial II, family members & SLTs)	*More understanding, patience, tolerance, empathy* *Feeling closer and more connected to each other* *Maintaining family roles and PWA taking on more responsibilities* * *more honest with each other* (partial II, PWA & family members) *less arguments and tension (partial II PWA & SLTs)

Abbreviations: APT, Aphasia Partnership Training; PWA, people with aphasia; SLT, speech and language therapist.

### Conversation

Stakeholders agreed on five items/aspects of ‘conversation’ that change was expected and likely in relating to awareness of communicative needs, thinking about use of different helpful ways of communication, comprehension, frequency and quality of conversations. Items with partial agreement reinforce the quality and use of strategies in conversation but also identify putting in effort or not giving up as important outcomes.

### Thoughts and feelings

All stakeholder groups agreed that *reduced frustration* was an important outcome of APT, with the highest number of combined votes (31) across PWA and family members (Appendix 2, Table 1c). Table 4 also shows that all stakeholder groups also agreed or partially agreed on a range of positive thoughts and feelings and a reduction in negative thoughts and feelings as a result of APT.

### Relationships

Table 4 shows that changes in ‘relationships’ following APT agreed upon by all stakeholder groups focus on being more understanding, patient and empathetic; being closer and more connected; and the PWA taking on more responsibility. All of the items identified by two of the three stakeholder groups in Table 4 were identified by either the PWA and SLTs or the family members and SLTs with the exception of being *‘more honest with each other’* where the agreement was between PWA and family members but not SLTs.

Table 4 presents the summary item labels that represent items with full and partial agreement across stakeholder groups for the constructs important to at least one stakeholder group. Wording of items and ratings given by each stakeholder group can be found in Appendix 2.

### Doing things items

Although change in the construct of ‘doing things’ wasn't of highest importance for any stakeholder group, they did identify some areas of change they partially agreed would be welcome, see Appendix 2, Table 1e. All three stakeholder groups would like to see change in *‘Doing more things with family and friends’* although SLTs did not reach consensus on how likely this was to change as a result of APT (partial agreement (I)). PWA identified that they would like to *‘do things more independently’* which was also recognised by SLTs (partial agreement II)). Family members wanted to be able to *‘make decisions and plans together’* which was also recognised by SLTs (partial agreement (II)). PWA and family members (but not SLTs) both wanted to *‘be more spontaneous’* (partial agreement II)).

### Other things

Two items with agreement from two or more stakeholder groups did not fit into any of the outcome constructs. *‘Raising awareness of aphasia’* as an outcome of APT was of particular importance to FMs receiving 30 votes and had full agreement across stakeholder groups. Some PWA hoped for *‘more acceptance of aphasia’* which was also recognised as a potential APT outcome by SLT (Appendix 2, Table 1f).

## DISCUSSION

This paper presents important outcomes of dyadic CPT for PWA and family members through the lens of improvements PWA and family members would hope to make if they received the APT intervention and the improvements SLTs would expect to see.

It was very important for all three stakeholder groups that APT should improve outcome constructs of ‘conversation’ and ‘thoughts and feelings’, with ‘conversation’ being the more important of the two. This is consistent with the finding of Wallace et al. (2017b) eDelphi with SLTs where very high levels of consensus (97%–99%) were achieved for outcomes relating to communication between the person with aphasia and their communication partner/s. Items illustrating what stakeholders mean by improved conversation in relation to APT are focussed on use of strategies by PWA and family members and improved communication skills and awareness of family members. They also focus on having ‘better’ conversations in terms of frequency, range of topics, depth and equal participation, spanning both activity and participation domains of the ICF. These items were also identified in the improved communication theme reported by Wallace et al. (2017a) in their identification of what PWA and family members would most like to change about their communication and the way aphasia affects their life. We reflect that the items within the construct of ‘conversation’, described communication more broadly than simply conversation in keeping with Wallace et al. (2017b, 2017a) and we will therefore call the construct ‘communication/ conversation’ when using these outcome constructs to complete the APT programme theory. Conversation as an outcome from aphasia rehabilitation is popular and a recent systematic scoping review identified 211 different ways of measuring conversation outcomes from across 64 studies (Azios et al., 2022). The current findings augment this array of metrics which focus on traditional linguistic analytical measures, conversation analytical measures, rating scales and others, and provide a unique insider perspective into what matters in conversation. Future work could explore the relationship between researcher‐defined and user‐defined outcomes in conversation.

Improvement in the construct of ‘thoughts and feelings’ was characterised by reduced frustration as the most important feeling to improve from APT for all stakeholders. Frustration is a pervasive consequence of aphasia reported by PWA and FM globally (Blom Johansson et al., 2012; Croteau et al., 2020; Wallace et al., 2017a) and emotional overwhelm and distress in carers is well documented (Luker et al., 2017); reducing this is an important outcome from intervention. Reducing frustration was also specified in Wallace et al. (2017a). Other improvements in thoughts and feelings included feeling good, happy, fulfilled, confident, relaxed and to have reduced negative emotions, all of which resonate with the category of ‘having more positive feelings’ within the construct of ‘improved emotional well‐being’ in Wallace et al. (2017a). SLTs are well attuned to the well‐being needs of PWA (Northcott et al., 2017) and internationally agree that improved mood, coping, and acceptance and reduced frustration and burden are priority outcomes from aphasia rehabilitation (Wallace et al., 2017b).

Improvements in the construct ‘talking (language)’ were the most important outcome from APT for PWA, but in stark contrast family members did not agree that changes in ‘talking (language)’ were important. This differs from Wallace et al. (2017a) work, in which *both* PWA and family members felt that it was important for the PWA's language function to improve. In addition, the language changes desired by PWA from APT focus on expressive language and word finding in particular, whereas Wallace and colleagues’ participants highlighted the desire to improve *all* language domains. PWA may have considered that they would ‘be able to speak better and find words more easily’ in the context of conversation where APT has enabled family members to give them more time in conversation to express themselves verbally. In contrast, it may be that, in valuing their involvement in the APT intervention, family members particularly focussed on dyadic communication and change in communication behaviour for both parties, making change in the PWA's talking/language less of a priority. Additionally, the finding may highlight a lack of awareness of the impact that family members have on PWA in conversation in supporting greater ability to express themselves verbally. In contrast to SLT views that it was important for language domains to improve with speech and language therapy in Wallace et al. (2017b), SLTs did not reach consensus about how likely language was to change from APT. It may be that, in keeping with the ICF domain of impairment, SLTs were considering improvement in language on a comprehensive language assessment, that is, outside of the context of conversation. However, in their paper on aphasia treatment approaches related to the ICF, Galletta and Barrett (2014) considered that supported conversation approaches may provide impairment‐based support (language improvement identified irrespective of context) as well as functional support. If future evidence of CPT outcomes supports this hypothesis, it would be beneficial to raise SLT awareness of the potential for CPT to improve language.

Improvement in relationships was very important to family members and SLTs and important to PWA, characterised by more understanding, patience, tolerance, empathy with one another, feeling closer and more connected and maintaining family roles. Relationships and connection with family and others are critical as they are core to living well and to quality of life with aphasia, with the centrality of communication to relationships explicit (Cruice et al., 2010; Ford et al., 2018; Manning et al., 2019). Relationships as a construct has more prominence in this study than in Wallace et al.’s (2017a) work where these ideas are represented, but ‘to participate in family relationships’ is a *category* within a theme for family members only, rather than being a *construct* in its own right for both PWA and family members. Impact on relationships also arose as a *subcategory* rather than a *theme* for SLTs in Wallace et al. (2017b). This difference may reflect APTs specific focus on the family dyad.

Although ‘doing things’ (ICF participation) did not eventuate as a consensus important outcome construct from APT for stakeholders, doing more things with family and friends would be welcomed as a result of APT. Stakeholders anticipated they could be doing more things at home or out and about including trying out new activities together or returning to previous activities, hobbies and interests; PWA also considered activities with friends. Doing ‘things’ or activities, having activities to do and having the ability to do activities are the foundation of living successfully with aphasia and contributes to current and future life quality with aphasia (Brown et al., 2010; Cruice et al., 2010). Further work is needed to explore and understand how for example improved conversation/communication might lead to increased life participation: is it through increased self‐confidence and self‐efficacy, or clearer articulation of desires?

Improvements that stakeholders hope for as outcomes of APT dyadic CPT have been discussed in relation to what they would generally like to change about communication, and the impact of aphasia reported by Wallace et al. (2017b, 2017a) which informed the Core Outcome Set (COS) for aphasia (Wallace et al., 2019). The reason for contextualising our results in this way was to understand whether desired outcomes from dyadic CPT are similar or different to the improvements desired generally. The fact that the outcome constructs and items reported in our study are also represented in Wallace et al. (2017b, 2017a) supports the face validity of our data, whilst at the same time, outcome areas which need specific focus from APT as we develop it have been identified. Our intention was not to develop a COS specifically for dyadic CPT or APT, thus we did not follow the COS standards for development (Kirkham et al., 2017). Rather, this work will help us to understand how to prioritise aphasia COS measures and identify additional measures required in the future evaluation of APT according to consensus on what is most important to achieve from dyadic CPT specifically.

### Strengths and limitations of the study

By using adapted nominal group technique methods we included the views of PWA with mild to severe aphasia in this study as well as family members and SLTs. In addition, we included the views of participants from a range of different ethnic groups. Sixty five percent of PWA were White British. In 2017, 88.4% of patients admitted to hospital with stroke in the United States were White British (Royal College of Physicians, 2017). It is known that stroke risk is more than twice as high in Black African than White British populations and they were represented in our sample (20%), although still underrepresented given the stroke risk (Ali et al., 2021). Unfortunately, other high‐risk groups for example, Asian/Pakistani were not represented, and family members were all White British.

A further strength of this study design was the addition of the perspectives from SLTs to the stakeholder triangulation as a moderator of the views of stakeholders who are potential intervention recipients. This enabled the importance of outcomes to be moderated by perceived likelihood of outcomes from the specific intervention. A key strength in generating items and summary labels was the reflexivity within the research team, assessment of researcher agreement, and modified member checking with the PPI group to ensure credibility of the resulting item labels.

Conducting consensus for PWA, family member and SLT groups separately and combining these data through triangulation only may be seen as a weakness of this work as the groups were unable to consider or vote on the views of one another resulting in a greater likelihood of silence in some constructs and items (considered by only one group). However, thoughts were not shared between groups as time constraints demanded that the nominal groups and eDelphis were run in parallel. More importantly, sharing views may have increased the complexity of the task for PWA and would have potentially diluted the raw patient perspective. A further weakness is that data on whether the participants had received CPT were not formally collected. However, anecdotally, we understand that the majority of participants had not experienced CPT and therefore they were suggesting improvements they would hope for based solely on a description of APT. Imagining outcomes of an intervention that has not been experienced may be more challenging, particularly for PWA. Reliance on an intervention description alone may therefore have led to different responses than if they had been informed by their own experiences of a different approach to CPT. Finally, SLT perspectives were limited by a relatively small number of participants completing the eDelphi.

### Implications of the study findings and next steps

These findings about what it is important and likely for APT to achieve can inform SLT and family dyad discussions of goals for family CPT programmes. The knowledge about expected changes from APT will also inform research questions when evaluating the effectiveness of the intervention and the choice of outcome measures to answer these questions. This information will be used to complete a programme theory for the APT intervention and to identify outcome measures whose items map most closely to those our stakeholders agreed are important.

### Conclusion

The most important outcomes of PWA and family member dyadic CPT based on a description of the APT programme are improvements in ‘conversation/communication’ and ‘thoughts and feelings’. Communication improvements should specifically include use of strategies by PWA and family members, improved communication skills and awareness of family members, plus ‘better’ conversations in terms of frequency, range of topics, depth and equal participation. Stakeholders characterised improvements in their thoughts and feelings as reduced frustration and feeling good, happy, fulfilled, confident and relaxed with a reduction in negative emotions about their communication. Improvement in relationships should be a key outcome of APT and was of particular importance to family members. PWA hope that APT would also improve their talking, specifically the ability to speak better and find words more easily. Outcomes expected from APT had considerable synergy with outcomes identified in previous work as being expected from speech and language therapy for aphasia in general, but with greater focus on relationships and a reduced focus on language outcomes. The examples of outcome constructs identified in this work illustrate what the outcomes mean to stakeholders in the context of APT dyadic CPT for PWA and family members.

## CONFLICT OF INTEREST STATEMENT

The authors declare no conflicts of interest.

## PARTICIPANT CONSENT STATEMENT

All participants provided informed consent.

## Supporting information



Online Appendix

## Data Availability

The data that supports the findings of this study are available in the supplementary material of this article.

## Footnotes

1 Five further family members consented to the study but were unable to attend the nominal groups.
