# Supplementary material for: Aphasia partnership training: What outcomes do people with aphasia, family members and speech and language therapists expect?
Source: Int J Lang Commun Disord. 2025 Feb 20;60(2):e70015. doi: 10.1111/1460-6984.70015 (PMC11842014; doi:10.1111/1460-6984.70015)
Supplement: Supplementary file 1 — Online Appendix [file JLCD-60-0-s001.docx]

Appendix 1 APT description

**What is APT?**

APT is a **new therapy** designed to help **people with aphasia** and their **family members communicate better together**. This training will always be delivered by a **qualified speech and language therapist.**It may be supported by **therapy assistants** or speech and language therapy **students**.​

**What might you do in APT?**

You and your family member might:

1. Discuss **tips for communicating** supported by the speech therapist.
2. **Find ways around communication problems.**
3. **Explore new ways of communicating** (the therapist will **show you how** to do these)
4. **Watch conversations of other people with aphasia and family members.** See **how** they communicate together. Think about whether any of the things they do might **work for you**.
5. Choose **what you’d like to focus on** in the communication therapy. Think about **what changes you hope to see**. **Agree on therapy goals.**
6. **Review your goals** with the therapist. Talk about **how you’re doing**. Decide whether you’d like to **stick with or change your goals.**
7. **Video record a conversation**. **Watch the conversation back** with the therapist. **Discuss how you are communicating** and **how you feel** about it.
8. Get **feedback** from the speech therapist about **what is going well.**
9. Discuss with the therapist **what happens** when you do certain things. Discuss **what might happen** if you tried something else when communicating.
10. **Practise new ways of communicating at home** and when you are **out and about.**
11. **Consider changing your surroundings. Consider bringing in new things** to help you communicate.
12. **Form new habits**. This will make doing new things when you are communicating **easier**.
13. **Receive** **support** from others.

These are the **main activities…** but there are **other things** **in the APT training** that can be recommended to improve communication between family members.

**Everyone is different. Every relationship is unique**. You will be given **lots of options** to choose from. See what you’d like to try and what works for you. **You decide** what good communication looks like for the two of you.

Appendix 2 Triangulation of outcome items

Table 1a) Items illustrating the ‘talking/language’ construct

| **LANGUAGE** | | | | |
| --- | --- | --- | --- | --- |
| **PWA**  **(number of votes)** | **Family members**  **(number of votes)** | **SLTs/Researchers**  **(no) consensus reached)** | **Convergence assessment** | **Summary statement** |
| My speech would be better  *(31)*  I would say what I want to say, and find my words more easily  *(21)*  I would be able to get my message across more clearly  *(14)*  I would be able to speak more quickly *(7)* | My relative with aphasia would find the right word more easily  *(2/6 participants voted)* | Improvements in PWA's speech production  (no consensus reached)  Improvements in PWA's language production  (no consensus reached)  Improvements in PWA’s word finding abilities  (no consensus reached) | **Partial agreement (I)** | **The person with aphasia would be able to speak better/find their words more easily** |
| --  -- |  | Improvements in PWA's language comprehension (inconclusive) | **Silence** | N/A |

Table 1b) Items illustrating the ‘conversation’ construct

| **CONVERSATION** | | | | |
| --- | --- | --- | --- | --- |
| **PWA**  **(number of votes)** | **Family members**  **(number of votes)** | **SLTs/ Researchers**  **(no) consensus reached)** | **Convergence assessment** | **Summary statement** |
| My family and other people would do things that help us to communicate better together  e.g. they would stop and give me time to talk  e.g. they would slow down when they speak  e.g. they would say one thing at a time  *(42)*  My family and others would stop doing things that do not help our conversation e.g. they would stop finishing my sentences   *(9)*  My family would listen to me when I talk  (4) | I would know how I can support their communication and we would use different ways to communicate that help make our conversation better  *(7)* | Both individuals in the family dyad become more skilled communicators who use more facilitative communication strategies/ reduce the use of behaviours that do not support conversation  (consensus reached)  The family dyad share information (e.g. communication needs/ helpful strategies) with others so those people can better support the person with aphasia in conversation  (consensus reached)  *AND*  Use of communication strategies becomes more instinctive/ automatic (consensus reached) | **Agreement** | **My family member, other people and I will use different ways of communicating that help conversation. We will be more mindful of the things we do in conversation, and work towards reducing behaviours that do not support conversation** |
| My family and others would know what I find hard, recognise how much effort I am putting in, and know how to communicate with me   *(26)* | I would know when to step in to help and when to hold back – I would give my relative with aphasia more opportunities to help themselves  *(7)* | More awareness of their own and/or the other person's communication needs, and a better understanding of how to reveal communicative competence  (consensus reached)  The family dyad share information (e.g. communication needs/ helpful strategies) with others so those people can better support the person with aphasia in conversation (consensus reached) | **Agreement** | **Being aware or recognising the needs of self and/or of the communication partner and knowing how to communicate better together.** |
| We would communicate more often   *(16)*  People would come and talk to me at family gatherings or social events *(2)*  I would talk to more people when I’m out and about, including to people I don’t know  *(2)* | My relative with aphasia would have more conversations and interactions with me and other people  *(7)* | The family dyad have more conversations  (consensus reached)  The person with aphasia has conversations with more people, which may include conversations in group contexts  (no consensus reached) | **Agreement** | **Communicating more often together/with others** |
| I would understand more of the conversation  *(13)*  My family and others would understand me better  *(5)*  I would not need to ask other people to repeat what they are saying as many times  *(3)* | We would understand each other better  *(6)* | Better able to understand what the other person is communicating  (consensus reached)  Less communication breakdowns/ fewer misunderstandings  (consensus reached) | **Agreement** | **Understanding more of the conversation/ understanding each other better** |
| We could have decent conversations where I say more than “yes”, “no”, or “maybe”  *(11)*  Our conversations would be more balanced and we could just chat   *(10)*  I would get my turn in the conversation  *(7)*  I would take part fully in conversations   *(6)* | Our conversations would be more interesting – they would be more than question and answer sessions  *(6)*  Our conversations would be longer and more in-depth  *(6)*  We would talk about a wider range of topics e.g. our plans for the future  *(6)* | More meaningful information is exchanged during conversations  (consensus reached)  Able to discuss more than basic needs, including more complex topics or communicating for a wider range of purposes e.g. expressing opinions/ discussing emotions (no consensus reached)  Conversations are longer/ more in-depth/ cover a wider range of topics  (no consensus reached)  More balanced conversation, including more equal contributions/ turn-taking (consensus reached)  The family conversation partner involves the person with aphasia more in the conversation, e.g. encouraging them to contribute/ not speaking for them (consensus reached)  The person with aphasia is more willing and able to initiate conversation/ choose the topic (no consensus reached)  Finding conversations more satisfying/ interesting/ enjoyable  (consensus reached) | **Agreement** | **Having a ‘decent’, more interesting or more balanced conversation** |
| I’d get better and faster at finding ways around problems e.g. I would practise drawing, or practice using my phone to help me when I get stuck in a conversation  *(10)*  Our conversation would flow more easily  *(2)* | -- | More effective and efficient repairs to conversation breakdowns e.g. shorter repair sequences (consensus reached)  Increased acceptance of non-verbal responses/ the use of total communication in conversation (consensus reached) | **Partial agreement (II)** | **Using better and faster ways of communicating (inc. non-verbal) to repair conversation breakdowns** |
| We would discuss everything, not just the happy or simple things  *(2)* | When we haven’t understood each other, we would be honest about this, and not pretend that we have  *(5)* | Conversations are more open and honest (no consensus reached) | **Partial agreement (I)** | **More open and honest conversations** |
| -- | Our conversations would be more light-hearted *(2)* | Conversations are more humorous (no consensus reached) | **Partial agreement (II)** | **Conversations are more light-hearted/humorous** |
| I would plan to have chats with others  *(3/5 hands raised)* | ***--*** | Less avoidance of situations which involve communicating (consensus reached) | **Partial agreement (III)** | **Making a deliberate effort to take part in conversation** |
| -- | My relative with aphasia would keep trying to communicate and not give up as easily *(1/4  hands raised)* | More resilient e.g. less likely to give up when encountering communication difficulties (consensus reached) | **Partial agreement (II)** | **Family dyad doesn’t give up when conversation gets hard** |
| -- | -- | Optimising the environment to facilitate better conversation (consensus reached) | **Silence** | **N/A** |
| -- | -- | Creating more opportunities to communicate together/ with others  (consensus reached) | **Silence** | **N/A** |
| -- | -- | Conversations are more similar to how they used to be before the stroke (no consensus reached) | **Silence** | **N/A** |

Table 1c items illustrating the thoughts and feelings construct

| **PWA**  **(number of votes** | **FM**  **(number of votes)** | **SLTs/ Researchers**  **(no) consensus reached)** | **Convergence assessment** | **Summary statement** |
| --- | --- | --- | --- | --- |
| I would feel less frustrated  *(15)* | Everyone would feel less frustrated  *(16)* | Feeling less frustrated (consensus reached) | **Agreement** | **Reduced frustration** |
| We would feel good   *(15)*  We would feel more relaxed  *(9)*  I would feel happy and fulfilled  *(7)* | Everyone would feel more confident  *(10)* | Feeling more confident in their abilities to communicate/ support communication (consensus reached)  Greater self-esteem/ self-belief (consensus reached)  Feeling more comfortable when using communication strategies (consensus reached)  Feeling more motivated/ empowered/ hopeful/ optimistic (consensus reached)  Feeling more competent/ that others recognise their competencies (consensus reached)  Feeling proud of themselves for trying different ways to make communication better (consensus reached)  Better mood (no consensus reached) | **Agreement** | **Feeling good, more relaxed, happy, fulfilled, free to be myself, more confident, comfortable, motivated, empowered, hopeful, optimistic, more competent** |
| I would feel less angry  *(1)* | We would try to take a step back and calm down to manage any negative emotions better e.g. when we are feeling angry or resentful  *(11)* | Feeling less resentful/guilty/ashamed about experiencing communication difficulties/negative emotions (consensus reached) | **Agreement** | **Feeling less angry, resentful, guilty** |
| I would feel less overwhelmed  *(1)* | I would feel more able to cope with my responsibilities  *(4)* | Feeling less helpless (consensus reached) | **Agreement** | **More able to cope** |
| I would feel more “normal” in conversations  *(3)* | *--* | Feeling more like themselves again (no consensus reached) | **Partial agreement (II)** | **Feeling more normal/like themselves again** |
| I would feel less panicky when I’m out and about  *(2)* | *--* | Feeling less anxious (no consensus reached) | **Partial agreement (II)** | **Reduced anxiety** |
| *--* | I would feel less lonely  *(4)* | Feeling less isolated/ lonely (no consensus reached) | **Partial agreement (II)** | **Reduced loneliness/isolation** |
| *I would feel less confused*  *(2)* | -- | -- | **Silence** | **N/A** |
| *I would feel less tired*  *(1)* | -- | -- | **Silence** | **N/A** |
| *--* | -- | Understanding that successful communication can take place without previous communication abilities being fully restored e.g. "My communication is good enough" or "Although it wasn't perfect, I understood what they were saying" (consensus reached) | **Silence** | **N/A** |
| *--* | -- | Trusting that the other person is doing their best too (consensus reached) | **Silence** | **N/A** |
| *--* | -- | Feeling like the responsibility to keep the conversation going or repair any breakdowns is more evenly shared (consensus reached) | **Silence** | **N/A** |
| *--* | -- | Aligning more to a social model of disability instead of a medical one e.g. understanding that language practice may not be the most effective way to make communication better (no consensus reached) | **Silence** | **N/A** |

Table 1d Items illustrating the ‘relationships’ construct

| **RELATIONSHIPS** |  |  |  |  |
| --- | --- | --- | --- | --- |
| **PWA** | **Family members** | **SLTs/ Researchers** | **Convergence assessment** | **Summary statement** |
| We would know each other better and be more understanding of each other  *(25)*  My family and others would be more tolerant of me and more patient with me  *(7)* | We would be more understanding of each other  *(5)* | A better understanding of each other (consensus reached)  More empathy/ patience/ respect for each other (consensus reached) | **Agreement** | **More understanding, patience, tolerance, empathy** |
| We would be honest about our situation and about the changes in our relationship  *(7)* | We would be more honest with each other *(2)* | -- | **Partial agreement (II)** | **More honest with each other** |
| We would feel closer together  *(4)* | We would have a more loving relationship  *(8)*  We would try to always stay friends  *(7)* | A stronger relationship: feeling closer/ more connected to each other (consensus reached)  More humour/ fun/ love in the relationship (no consensus reached) | **Agreement** | **Feeling closer and more connected to each other** |
| I would resume my role(s) in the family and my family would share things with me e.g. their troubles as well as their triumphs  *(4)* | We would make decisions together *(3)* | The relationship is more balanced/ equal e.g. the person with aphasia taking on a more active role/ the family conversation partner feeling less burdened (consensus reached) Maintaining family roles (no consensus reached) | **Agreement** | **Maintaining family roles and PWA taking on more responsibilities** |
| We would accept that changes are needed on all sides (i.e. it isn’t down to one person to make all the changes) *(4)* | *--* | The relationship is more balanced/ equal e.g. the person with aphasia taking on a more active role/ the family conversation partner feeling less burdened (consensus reached)  Both supporting one another (i.e. support is two-way) (no consensus reached) | **Partial agreement (II)** | **Taking an active role and providing support to each other** |
| We would argue less  *(2)* | *--* | Less conflict/ tension/ strain (no consensus reached) | **Partial agreement (II)** | **Less arguments and tension** |
| -- | *--* | A bigger social circle which may include new connections (no consensus reached) | **Silence** | **N/A** |

Table 1e items illustrating ‘doing things’ construct

| **DOING THINGS** |  |  |  |  |
| --- | --- | --- | --- | --- |
| **PWA** | **Family members** | **SLTs/ Researchers** | **Convergence assessment** | **Summary statement** |
| We would do more things together at home and/or out and about e.g. walking the dog, gardening, shopping, going for nights out  *(21)*  We would go out more with our family  *(1)*  I would go out and do things with my friends  *(16)* | We would go out and do things (trying new activities or returning to previous hobbies or pursuits)  *(15)* | Doing more things around the home or when out and about (together/ alone/ with others) (no consensus reached)  Taking part in more leisure activities (trying new ones and/or returning to previous ones) (no consensus reached)  Being more social/ engaging with a wider social network (no consensus reached) | **Partial agreement (I)** | **Doing more things with family and friends** |
| I would do more on my own e.g. exercising, hobbies, or volunteering *(2)*  I would try to get back to normal. This might include going back to work, or managing my own affairs (e.g. my banking) *(1)* | -- | Taking part in more leisure activities (trying new ones and/or returning to previous ones) (no consensus reached)  The person with aphasia is more independent in everyday activities (no consensus reached)  Returning to work (no consensus reached)  Doing more in the community e.g. accessing local services (no consensus reached) | **Partial agreement (II)** | **PWA does things more independently** |
| I would be more spontaneous and do things without having to stop and think too much *(2/4 hands raised)* | We would be more spontaneous and do things on a whim  *(3/4 hands raised)* | -- | **Partial agreement (II)** | **Being more spontaneous** |
| -- | We would discuss and make plans for the future in case something happens to one of us e.g. sorting out our wills and power of attorney  *(4/4 hands raised)* | The person with aphasia is more involved in decision-making, e.g. when planning things to do | **Partial agreement (II)** | **Making decisions and plans together** |
| I would be able to help other people in a similar situation  (5) | -- | -- | **Silence** | **N/A** |

Table 1f items illustrating the ‘other things’

| **OTHER THINGS** | | | | |
| --- | --- | --- | --- | --- |
| **PWA** | **Family members** | **SLTs and Researchers** | **Convergence assessment** | **Summary statement** |
| I would raise awareness about aphasia: I would tell more people about it, and share tips on how to talk to someone with aphasia  *(12)* | More people would know about aphasia, and have a better understanding of how stroke and aphasia can change lives  *(30)* | More knowledge/ insight/ awareness of aphasia and its impact (consensus reached) | **Agreement** | **Raising awareness of aphasia** |
| I would come to terms with having aphasia  *(4)* | -- | More acceptance of living with aphasia | **Partial agreement (II)** | **More acceptance of aphasia** |
